# Do Ecological Restoration Projects Improve Water-Related Ecosystem Services? Evidence from a Study in the Hengduan Mountain Region

**DOI:** 10.3390/ijerph19073860

**Published:** 2022-03-24

**Authors:** Le Yin, Shumin Zhang, Baolei Zhang

**Affiliations:** 1 College of Geography and Environment, Shandong Normal University, No. 1, University Road, Changqing District, Jinan 250358, China; yinl.16b@igsnrr.ac.cn; 2 Institute of Regional Economic Research, Shandong University of Finance and Economics, Jinan 250014, China; zhangshumin999@163.com

**Keywords:** water yield, soil erosion, LULC, climate change, trade-offs, Hengduan Mountain region

## Abstract

Land use/land cover (LULC) and climate change are major driving forces that impact ecosystem services and affect human well-being directly and indirectly. Under the future interaction between LULC and climate change, the impact of different land management and climate change scenarios on water-related services is uncertain. Based on this, the CLUMondo model, which focuses on land use intensity, was used to simulate the land system under different land management scenarios in the future. By coupling the downscaled climate scenario data, this study used the InVEST and RUSLE models to estimate the annual water yield and soil erosion in 2050 in the Hengduan Mountain region and analyzed the variation differences in different sub-watersheds. The results indicated that, under the influence of LULC and climate change, when compared with the amount for 2020, the soil erosion in the Hengduan Mountain region in 2050 was reduced by 1.83, 3.40, and 2.91% under the TREND scenario, FOREST scenario, and CONSERVATION scenario, respectively, while the water yield decreased by 5.05, 5.37, and 5.21%, respectively. Moreover, the change in soil erosion in the study area was affected by precipitation and closely related to the precipitation intensity, and the impact of climate change on the water yield was significantly greater than that of LULC change. The spatial heterogeneity of soil erosion and water yield was obvious at the sub-watershed scale. In the future, soil erosion control should be strengthened in the northern regions, while water resource monitoring and early warning should be emphasized in the central-eastern regions. Our results provide scientific guidance for policy makers to formulate better LULC policies to achieve regional water and soil balance and sustainable management.

## 1. Introduction

Ecosystem services (ESs) are influenced by natural and human factors and can directly or indirectly affect human well-being [1,2]. By quantifying and mapping ESs, resources can be identified at the regional level, and scientific management can be realized [3]. In recent years, many ecological and environmental problems have arisen due to rapid population growth, accelerated urban expansion, and social and economic development, which pose serious threats to regional ESs [4,5]. The Millennium Ecosystem Assessment showed that approximately 60% of global ESs are and will continue to degrade, which will seriously affect regional ecological security and human well-being [6]. Therefore, understanding future trends of regional ESs is crucial for decision makers to scientifically manage ecosystems and ensure human well-being.

Land use/land cover (LULC) and climate change are recognized as two major driving forces affecting regional ESs [7]. LULC change is one of the main causes of global environmental change. LULC change alters the surface structure, affects the regional climate, hydrology, water resources, soil, biodiversity, and biogeochemical cycles and directly impacts the structure and function of entire ecosystem. LULC change impacts ESs in three main ways: (1) land-use type change; (2) land-use pattern change; (3) land-use intensity change. In recent years, with the increasingly prominent ecological and environmental problems in China, the Chinese government has carried out six national ecological restoration projects (ERPs) across the country, aiming to restore the damaged ecological environment [8]. The implementation of these ERPs has had a positive impact on the regional land-use structure, which is mainly reflected by a significant increase in ecological land (forestland, grassland, water area, etc.). For example, the ERPs not only significantly reduce the amount of soil erosion on the Loess Plateau but also decrease soil erodibility [9]; with the change of land use pattens, the water-related services in the Heihe River Basin have undergone irreversible changes, and the development of large-scale irrigated farmland and human activities are a source of further intensification of regional soil erosion and water pollution [10]. Thus, effective land-use management is the key to achieving the sustainable development of regional ESs [11,12]. In addition, climate change plays a decisive role in regional ESs. For instance, climate factors such as air temperature, precipitation, and sunshine are related to surface evaporation, transpiration, and soil moisture changes, which affect the water yield of basins and the regional hydrological budget [13]. As concerns about water scarcity and water quality degradation increase, research on water-related ESs can help policy makers address acute water challenges. Therefore, exploring the future impacts of LULC change and climate change on water-related ESs is of great significance for sustainable water resource utilization and ecological security.

A substantial amount of research has been conducted on the driving mechanism of water-related services. Previous studies have systematically revealed the interactions between ecosystems and influencing factors from different perspectives (e.g., socioeconomic and ecological), using various methods (e.g., correlation analysis, geographically weighted regression (GWR), and geo-detector) and based on different spatiotemporal scales (e.g., global, regional, watershed, historical, interannual, and annual). However, research has mainly focused on analyzing the driving mechanism of ESs in the past, and the evolution of ESs under different scenarios on a long-term scale in the future has not received enough attention [14]. Given the long-term characteristics of climate change and the lag after the implementation of ERPs, research on the impacts of ERPs and climate change on regional water-related services on a long-term scale is necessary [15,16]. Moreover, most studies have analyzed individual driving forces or services [17], and few studies have focused on the comprehensive impact of LULC change and climate change on multiple water-related services [18,19,20]. In addition, in areas with relatively stable land-use patterns, land-use intensity is the key driving force of regional ESs, but land-use intensity is often ignored in existing studies.

The Hengduan Mountain region is an important water conservation area in China, and one of the areas with the most serious soil erosion [21,22]. The optimal management of water-related services in the Hengduan Mountain region is related to regional sustainable development and water security in the lower reaches of the rivers there. The purpose of this study was to (1) simulate the future land system of the Hengduan Mountain region under the influence of land-use intensity and management measures; (2) explore the spatiotemporal evolution characteristics of water-related services under different scenarios; (3) reveal the trade-offs and synergies between different water-related services in the future. The purpose of this study was to provide a scientific basis for the sustainable utilization of regional water resources and watershed water security.

## 2. Materials and Methods

### 2.1. Study Area

**Figure 1:**
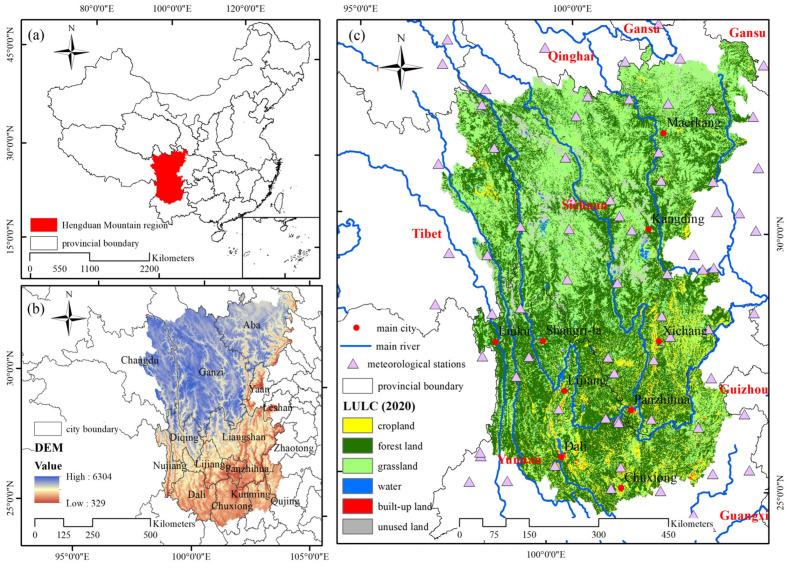
Basic information of the Hengduan Mountain region. (**a**) Location, (**b**) DEM, and (**c**) LULC map.

The Hengduan Mountain region (24°29′–33°43′ N, 97°10′–104°25′ E) is located in southwestern China and is famous for its complex terrain and obvious vertical climate changes. The Hengduan Mountain region covers an area of approximately 450,000 km^2^, with an average elevation of more than 3000 m. To protect the fragile ecological environment of the region, the forestland area of the Hengduan Mountain region has expanded significantly in the past 20 years under the influence of the Shelterbelt Project in the middle and upper reaches of the Yangtze River. The main land-use types in the Hengduan Mountain region are forestland, grassland, and cropland. Taking 2020 as an example, the forestland area accounted for 46.45% of the total area, the grassland area accounted for 41.24% of the total area, and the cropland area accounted for 7.71% of the total area (Figure 1).

### 2.2. Research Framework

**Figure 2:**
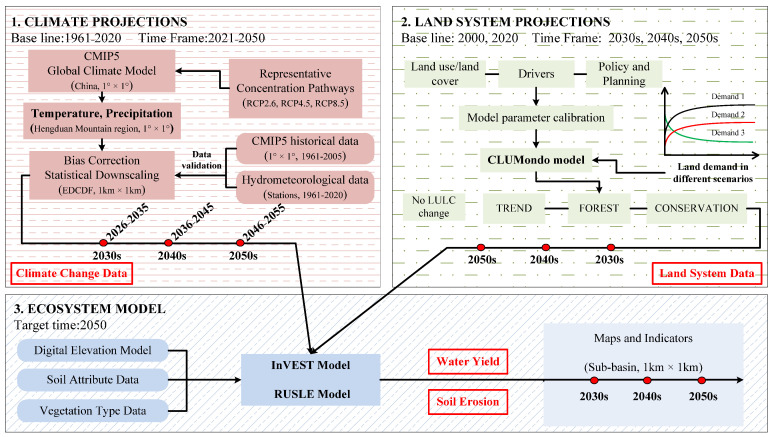
Framework of this study.

To explore the possible consequences of changes in climate, land use, and policy management practices, an integrated modeling approach was developed to assess future water-related services and their trade-offs under different scenarios (Figure 2). The integrated model included three main components, climate projections, land system projections, and ES assessment models. The climate data used in this study were CMIP5 multimodel coupled data provided by the National Climate Center, including long-term historical climate simulation and future climate scenario data from the following representative concentration pathways (RCPs): RCP2.6, RCP4.5, and RCP8.5. Land use/land cover data with a resolution of 1 km was obtained from the Resource and Environment Science and Data Center, Chinese Academy of Sciences. These data sources provide dynamic inputs to the ecosystem models (i.e., InVEST, RUSLE) and can be used to explore the likely consequences of various changes in management policies, climate, and land-use, and assess the trade-offs between water-related services (Table S1).

### 2.3. Land System Change Scenarios

Given the importance of the Hengduan Mountain region to the protection of the ecological environment of the region and even all of China, according to different development strategies, the no LULC change, TREND, FOREST, and CONSERVATION scenarios were established to explore the future land system development trajectories.

(1) No LULC change scenario.

This scenario was selected to describe the impact of climate change on future water-related services, especially water production services that are greatly affected by climate change. In this scenario, the base period data were the land system data in 2020.

(2) TREND scenario.

This scenario assumes a future situation in which land demand is steadily increasing and policies and measures remain unchanged. The TREND scenario follows the evolution rule of the land system in the Hengduan Mountain region in the past decade.

(3) FOREST scenario.

The implementation of the Shelterbelt Project in the upper reaches of the Yangtze River has significantly improved the vegetation conditions in the Hengduan Mountain region and limited the intensity of soil erosion. This study focused on the role of forests in regional soil and water conservation by setting up the FOREST scenario.

(4) CONSERVATION scenario.

The Hengduan Mountain region boasts some of the richest biodiversity in the world. The protection of regional biodiversity is of great importance to the ecological security of the country and even the world. This scenario simulates the evolution of the land system under the CONSERVATION scenario by taking nature reserves as restricted development areas and controlling the total amount of ecological land in the region.

**Figure 3:**
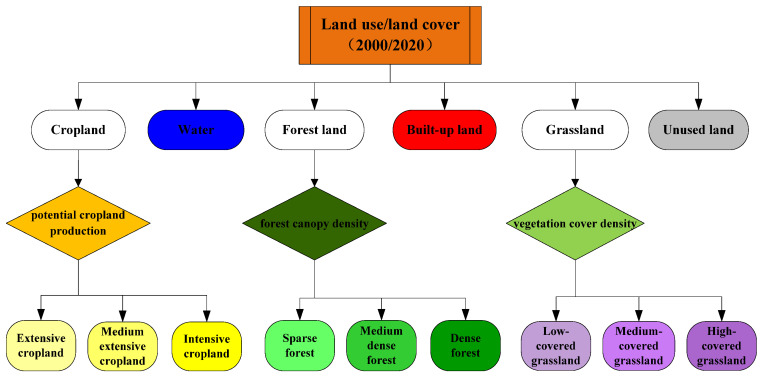
Approaches to land system delineation.

Land-use intensity and land management policy are important factors affecting the land system in mountainous areas. In this study, we selected the CLUMondo model, which was specifically designed to simulate changes in land-use cover and land-use intensity, to simulate changes in the land system under different scenarios in the Hengduan Mountain region. The CLUMondo model is a dynamic, spatial, and intuitive land-use model that takes the land system rather than the land cover type as the simulation unit. This innovative method can be used to simulate changes in regional land systems that are driven by the demand for various commodities or services. In this study, land use/land cover data from 2000 and 2020 were used as the base period. The cropland was divided into extensive cropland (1041.49 kg ha^−1^), moderately extensive cropland (4017.17 kg ha^−1^), and intensive cropland (>4017.17 kg ha^−1^) by using farmland potential productivity data; forestland was divided into sparse forest (37.01%), moderately dense forest (67.25%), and dense forest (>67.25%) by using forest canopy density data; grassland was divided into low-coverage grassland (46.27%), medium-coverage grassland (70.98%), and high-coverage grassland (>70.98%) by using vegetation coverage data (Figure 3) [21,23,24,25]. The supply of ESs per pixel was determined by the total supply of regional ESs, such as crop production. First, the number of pixels of extensive cropland, moderately extensive cropland, and intensive cropland was calculated to determine their proportional areas and then pixel-scale crop production levels were identified according to regional crop production levels. A total of 20 driving factors, including climate, topography, soil attributes, socioeconomic concerns, and vegetation types, were used as explanatory variables for changes to the land system in the Hengduan Mountain region. Constraints on the evolution of the land system under different future scenarios included crop production, livestock numbers, built-up land area, forestland area, ecological land area, and restricted areas (Table S2). In this study, the precision of the CLUMondo model was verified by the Kappa simulation coefficient, based on a comparison between the simulated data and the actual data of the land system in the Hengduan Mountain region in 2020 [26].

### 2.4. Statistical Downscaling of the RCP Scenarios

Scenarios of greenhouse gas emissions form the basis of projections of future climate change. The Intergovernmental Panel on Climate Change (IPCC) Fifth Assessment Report assessed 21st century projections under a new generation of greenhouse gas emission scenarios called RCPs. The RCP emission scenarios are based on a series of factors, such as population growth and economic development, and the categories include RCP 2.6 (low greenhouse gas emissions), RCP 4.5 and RCP 6.0 (intermediate greenhouse gas emissions), and RCP 8.5 (high greenhouse gas emissions). Currently, RCP emission scenarios are widely used to assess the impact of climate change on regional hydrology and water resources [27,28,29]. The climate change projection data used in this study are the “China Regional Climate Change Projection Dataset, Version 3.0”, generated by the National Climate Center based on CMIP5 multimodel data.

Projections of climate change are usually based on large global scales, meaning that data products cannot reliably provide predictions at local scales. To obtain climate projection data that can reflect regional differences, the GCM outputs were bias-corrected based on observation data by using the equidistant cumulative distribution function (EDCDF) method, and downscaled statistically to a regular geographical grid with a resolution of 1 km by using the statistical downscaling model (SDSM).

### 2.5. Water-Related ES Quantification

(1). Water yield.

Based on the water balance principle, the InVEST model simulates water yield on a pixel scale by calculating the difference between precipitation and actual evaporation. The formula is as follows [30]:

(1) $$Y_{\mathrm{xj}}=\left(1-{\mathrm{AET}}_{\mathrm{xj}}/P_{\mathrm{xj}}\right)\cdot P_{\mathrm{xj}}$$

where $Y_{\mathrm{xj}}$ is the annual water yield (mm) of land-use type j in grid x; ${\mathrm{AET}}_{\mathrm{xj}}$ is the annual mean actual evapotranspiration of grid x (mm); $P_{x}$ is the annual mean precipitation (mm) of grid x.

(2). Soil erosion.

Based on the revised universal soil loss equation (RUSLE) model, this study assessed the soil erosion rates for each grid. The formula is as follows [31]:

(2) SE=R×K×L×S×C×P

where SE is the annual soil erosion rate (t ha^−1^ yr^−1^); R is the rainfall erosivity factor (MJ mm ha^−1^ h^−1^ yr^−1^); K is the soil erodibility factor (t ha h MJ^−1^ mm^−1^ ha^−1^); L,S is the slope length–steepness factor; C is the cover management factor; P is the erosion control practice factor.

## 3. Results

### 3.1. Land System Changes under Different Future Scenarios

**Figure 4:**
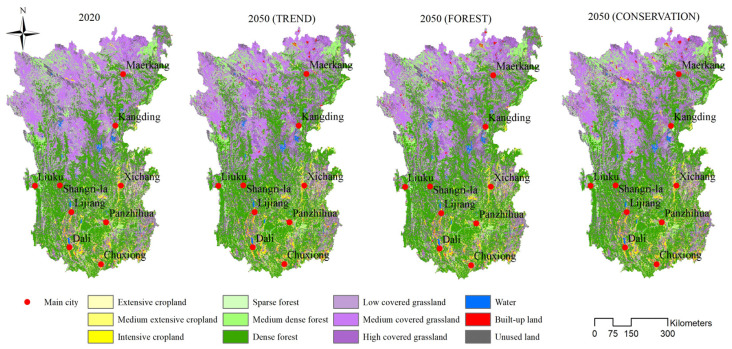
Simulation results of the land system in the Hengduan Mountain region in 2050.

**Figure 5:**
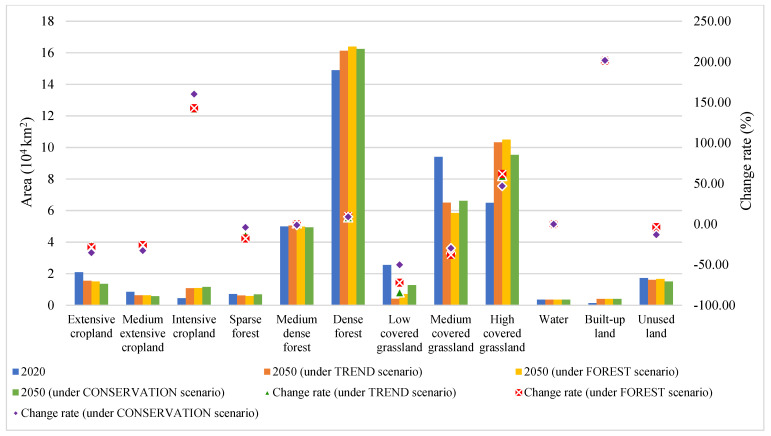
Variation of each land system type under different scenarios (2050) in the Hengduan Mountain region.

The overall accuracy of the CLUMondo model simulation was 0.84; the Kappa transition and Kappa location transition coefficients were 0.90 and 0.95, respectively. Under the three scenarios, forestland, grassland, and cropland still occupied the dominant positions, accounting for approximately 90% of the total area (Figure 4). In 2050, the land system change in the Hengduan Mountain region was mainly characterized by intensification, with cropland, forestland, and grassland being the most obvious. The areas of intensive cropland in the cropland system, dense forest in the forestland system, and high-coverage grassland in the grassland system increased significantly. However, under the influence of the implementation of ERPs, the area of forestland in the Hengduan Mountain region obviously expanded, while the area of grassland showed a trend of gradual decrease under different scenarios. In addition, the area of built-up land in the Hengduan Mountain region grew the fastest, roughly doubling by 2050, while water and unused land remained relatively stable (Figure 5).

### 3.2. Climate Scenario Data Correction and Its Variation Characteristics

Given that the historical period data provided by the CMIP5 multimodel dataset were available up to 2005, bias correction of the climate change scenario data was performed from the perspectives of temporal variation and spatial distribution, based on the actual observation data from 1961–2005. The results indicate that bias correction and statistical downscaling led to a significant improvement in the ability of the GCMs to reproduce the observed spatial pattern and long-term average of climatic variables. In the temporal dimension, the accuracy of the corrected simulation results was greatly improved after the bias correction of daily climate variables, whether on the interannual or inter-monthly scales (Figures S1 and S2). In the spatial dimension, the spatial correlation coefficient between the bias-corrected climate variables, and the observed climate variables was above 0.95 and passed the 0.01 level significance test (Figures S3 and S4).

We selected an intermediate climate change scenario (RCP 4.5), which can better reflect the current climate change trend, to assess the potential impact of ERPs on future water-related services. The results indicate that the annual mean temperature and annual precipitation in the Hengduan Mountain region showed an upwards trend from 2020 to 2050 under the RCP 4.5 scenario. When compared with 2020, the annual mean temperature and annual precipitation increased by 1.17 °C and 57.12 mm, respectively.

### 3.3. Scenario Analysis of ES Changes

(1). Soil erosion.

**Figure 6:**
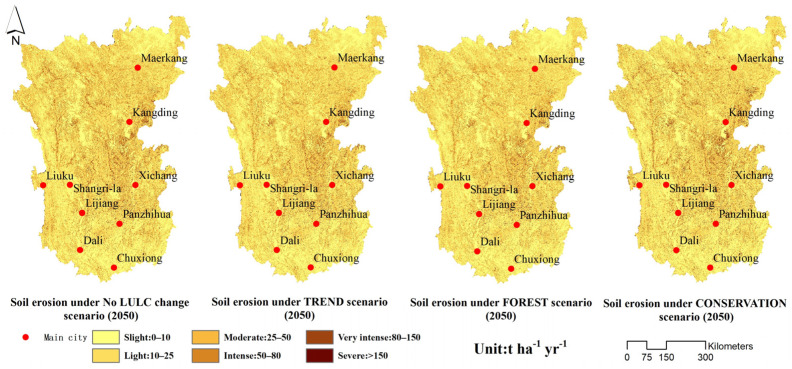
Spatial distribution of soil erosion under different scenarios (2050).

Figure 6 shows the distribution pattern of soil erosion in the Hengduan Mountain region. The results indicate that the distribution pattern of soil erosion in the Hengduan Mountain region did not change from 2020 to 2050. According to the Soil Erosion Classification Standard (SL190) issued by the Ministry of Water Resources, PRC, soil erosion was classified into six categories: slight (10 t ha^−1^ yr^−1^), light (25 t ha^−1^ yr^−1^), moderate (50 t ha^−1^ yr^−1^), intense (80 t ha^−1^ yr^−1^), very intense (150 t ha^−1^ yr^−1^), and severe (>150 t ha^−1^ yr^−1^). In 2020, the proportion of areas with slight, light, moderate, intense, very intense, and severe soil erosion in the study area was 38.76, 31.13, 18.53, 7.32, 3.53, and 0.73%, respectively. The regions with intense soil erosion (soil erosion >50 t ha^−1^ yr^−1^) were mainly distributed along the sides of the Lancang River, Jinsha River, Yalong River, and Dadu River.

**Figure 7:**
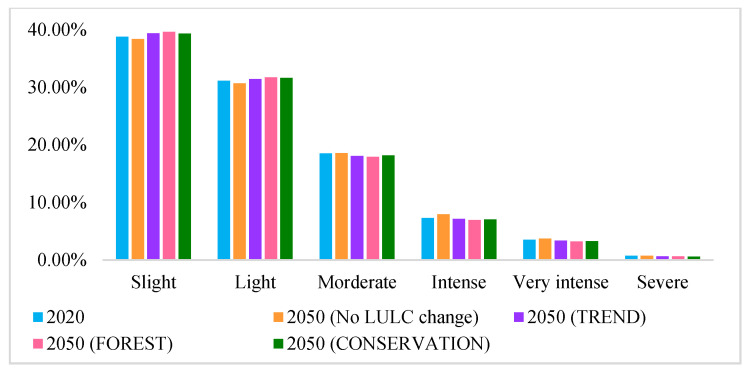
Percentage of soil erosion level under different scenarios (2050) and for 2020.

In 2050, the soil erosion values under the no LULC change scenario, TREND scenario, FOREST scenario, and CONSERVATION scenario in the Hengduan Mountain region were 22.86, 22.02, 21.67, and 21.78 t ha^−1^ yr^−1^, respectively. When compared with 2020, soil erosion in the Hengduan Mountain region in 2050 showed a decreasing trend under different scenarios, except for the no LULC change scenario. Soil erosion decreased by 1.83% under the TREND scenario, by 3.40% under the FOREST scenario, and by 2.91% under the CONSERVATION scenario. Moreover, in 2050, the total area with intense soil erosion, very intense soil erosion, and severe soil erosion declined under the three scenarios, from 11.58% in 2020 to 11.18% (TREND scenario), 10.77% (FOREST scenario), and 10.92% (CONSERVATION scenario), respectively (Figure 7). Therefore, the soil erosion was the smallest and had the smallest area under the FOREST scenario. An interesting finding was that under the no LULC change scenario, although precipitation decreased, the soil erosion in 2050 was higher than that in 2020. The precipitation erosivity in 2050 was significantly greater than that in 2020. Therefore, soil erosion is closely related to precipitation intensity [22,32,33]; that is, future climate change is more conducive to the occurrence of soil erosion [34,35].

(2). Water yield.

**Figure 8:**
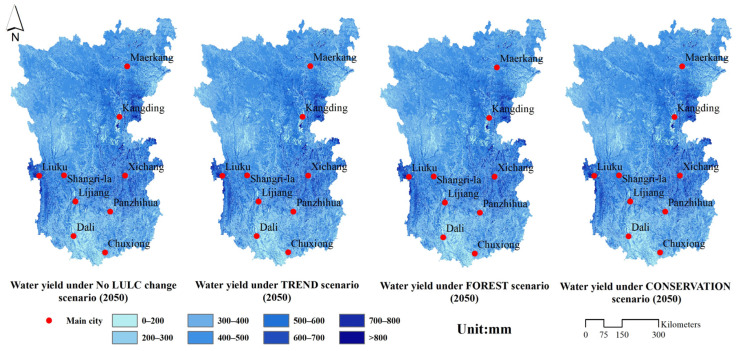
Spatial distribution of water yield under different scenarios (2050).

Figure 8 shows the distribution pattern of water yield in the Hengduan Mountain region. It should be noted that to explore the impact of climate change on water-related services, land data from 2020 were used for the water yield assessment under the no LULC change scenario. The results indicate that the areas with higher water yields in the Hengduan Mountain region under different scenarios (2050) were mainly distributed in the southwest Three Parallel River area and the middle-east Dadu River basin.

In 2020, the water yield in the Hengduan Mountain area was 423.79 mm. When compared with 2020, the water yield in the Hengduan Mountain area showed a decreasing trend under different scenarios (2050), among which the no LULC change scenario showed a decrease of 4.30%, the TREND scenario showed a decrease of 5.05%, the FOREST scenario showed a decrease of 5.37%, and the CONSERVATION scenario had a decrease of 5.21%. By comparing the change in the water yield under the scenarios without land change (no LULC change scenario) and scenarios with land system change (TREND scenario, FOREST scenario, and CONSERVATION scenario) during 2020–2050, climate change was found to be the main factor affecting water yield under different scenarios in the Hengduan Mountain region when compared with the evolution of the land system.

## 4. Discussion

This study focused on mountainous areas that have generally received less research attention and systematically analyzed the potential impact of ERPs on water-related ESs in the Hengduan Mountain region. The results of this study are consistent with the expectations of the Chinese government when implementing large-scale ERPs; namely, the implementation of ERPs could significantly inhibit regional soil erosion. The main reason is that forests are better at water and soil conservation than other vegetation types. However, the excessive pursuit of forest areas to reduce soil erosion can lead to grassland degradation and a sharp decrease in cropland. Because there is less cropland available in the Hengduan Mountain region, the excessive occupation of cropland may threaten the livelihood of residents [36]. Based on these results, the people’s governments of Sichuan Province, Yunnan Province, and Tibet Autonomous Regions issued strict cropland protection policies around 2018 to ensure regional food security. In addition, under conditions of limited human material and financial resources in the future, regional ecological restoration should be more refined. Within the allowable range of soil erosion, regionally differentiated ecological restoration strategies are indeed necessary [7,37]. For example, in areas with different vegetation coverage, soil properties, and water and soil conditions, artificial afforestation, natural restoration, or a combination of these methods can be adopted [38,39].

Although climate change was found to be the main factor affecting the water yield in the Hengduan Mountain region, the implementation of ERPs reduced regional soil erosion while simultaneously accelerating water yield declines. Many studies have shown that newly planted forests consume more water than native plants [40]. The Hengduan Mountain region is the birthplace and home of many domestic and international rivers. Its water resources are related to the sustainable development of this region and water security in the downstream areas. As the middle and lower reaches of the Yangtze River are facing serious water shortages due to uneven precipitation, population growth, and water consumption by industry and agriculture [41,42], a broader and more comprehensive assessment of hydrological processes is essential to ensure regional water security.

**Figure 9:**
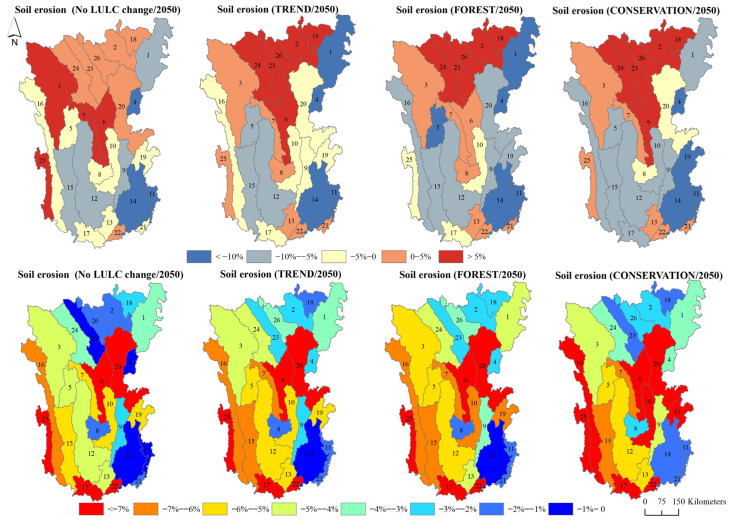
Change rate of soil erosion and water yield in the sub-watersheds under different scenarios (2050) when compared with 2020.

The most important problem in the Hengduan Mountain region is serious soil erosion, but with the implementation of the Yangtze River Shelter Forest Project, Natural Forest Protection Project, and Grain for Green Program, soil erosion has been well restrained [22]. However, consistent with the conclusions of many studies, this study found that there was an obvious trade-off between soil erosion and water yield; that is, regional soil erosion was reduced at the expense of water yield [43,44,45]. Given that water resources are not only related to the sustainable development of the regional ecosystem but also an important guarantee for the livelihood of the basin’s residents, many articles have suggested that ecological restoration strategies of large-scale afforestation should be modified [46,47]. Identifying the balancing point between the suppression of soil erosion upstream through ecological engineering and ensuring water security in the middle and downstream areas should be the key topic of follow-up research [48,49]. Moreover, studies have shown that the increase in the net primary productivity of vegetation due to vegetation restoration has a threshold effect [50]. When the vegetation coverage reaches a certain range, natural vegetation restoration is a better ecological restoration path than large-scale afforestation [51]. In addition, due to the spatial heterogeneity of the spatial distribution of soil erosion and water yield (Figure 9), the analysis of changes at the sub-watershed scale is essential for the accurate management of water-related services. The change rates of soil erosion and water yield under the four scenarios at the sub-watershed scale indicated that, under the influence of LULC change and climate change in the future, the soil erosion in the northern and central parts of the Hengduan Mountain region (sub-watersheds 2, 6, 18, 23, 24, and 26) will gradually increase, which can be restrained by strengthening the implementation of ERPs. Additionally, the sub-watersheds with greater water yield decline are located in the southwest and the middle-east (e.g., sub watersheds 6, 17, 20, 22, and 25), and the sustainable utilization of water resources can be ensured by strengthening water resource monitoring and early warnings in these sub-watersheds. The identification of regional priority restoration areas based on ecological quality, ecological health, and ESs can provide a scientific reference for the order of ecological restoration in different sub-watersheds [52].

Due to data acquisition limitations, only climate change and land system change were selected as the main driving factors of regional water-related services to conduct driving mechanism research. However, in reality, regional ecological restoration is a long-term and complex process, and anthropogenic, natural, soil attribute, and vegetation factors are not independent; rather, they are closely related and interactive [53,54]. Studies have shown that the implementation of ERPs can change soil attributes and reduce soil erodibility [55]. However, obtaining soil attribute data over long-term scales is challenging [56]. Higher quality, long-term data are an important guarantee for clarifying the impact mechanism of regional water-related services. Future research should focus on establishing a process model that can represent the interaction of various factors and coupling the multisource data of vegetation, soil attributes, and precipitation at different spatial scales [57].

This study assessed the potential impact of LULC change and climate change on regional water-related services under different scenarios, but the results may be uncertain due to the limitations of the data and methods. First, the model used in the assessment of water-related services in this study has some limitations. For example, the values of the parameters C and P in the RUSLE model were all taken from relevant literature in surrounding areas. The InVEST model does not distinguish between surface water, groundwater, and baseflow, nor does it consider their interactions. Second, global climate models are inherently uncertain due to their complexity. Although this study adopted an optimized statistical downscaling model to downscale climate variables, this approach might introduce new uncertainties. With the continuous improvement of spatial data accuracy, more monitoring sites, and more reliable biological-physical model simulation results, an accurate understanding of the regional resource background, ecosystem function and service status will be realized.

## 5. Conclusions

Based on LULC scenarios (no LULC change, TREND, FOREST, and CONSERVATION) and climate scenarios (RCP 4.5), this study assessed the water-related ESs in the Hengduan Mountain region and analyzed the spatial heterogeneity in different sub-watersheds. The results indicate that climate change is the main factor affecting water yield in the Hengduan Mountain region, and the implementation of ERPs can reduce regional soil erosion, while simultaneously accelerating water yield declines. When compared with the values for 2020, in different LULC and climate scenarios, the soil erosion and water yield will decrease the most, by 3.40% and 5.37%, respectively, under the FOREST scenario. At the sub-watershed scale, the northern part of the Hengduan Mountain region increased soil erosion in 2050, while the central-eastern regions showed obviously decreased water yields. The research results can provide methodological support for the comprehensive simulation of LULC and climate change impacts, and provide a scientific reference for the formulation of LULC policies for sustainable water resource development. The results also provide a methodological perspective to improve the accuracy and effectiveness of climate and LULC scenarios in ES evaluations.

## Data Availability

The data presented in this study are available on request from the corresponding author.

## Supplementary Materials

The following supporting information can be downloaded at: https://www.mdpi.com/article/10.3390/ijerph19073860/s1: Figure S1: Comparison between observed precipitation and temperature before and after model revision on an annual scale in the Hengduan Mountain region; Figure S2: Comparison between observed precipitation and temperature before and after model revision on monthly scale in the Hengduan Mountain region; Figure S3: Comparison of spatial distribution of precipitation in the Hengduan Mountain region before and after model revision; Figure S4: Comparison of spatial distribution of temperature in the Hengduan Mountain region before and after model revision. Table S1: Sources of data used in this study; Table S2: Growth rate of demand for land-use under different scenarios (%/year).
