# Supplementary material for: Do Ecological Restoration Projects Improve Water-Related Ecosystem Services? Evidence from a Study in the Hengduan Mountain Region
Source: Int J Environ Res Public Health. 2022 Mar 24;19(7):3860. doi: 10.3390/ijerph19073860 (PMC8997630; doi:10.3390/ijerph19073860)
Supplement: Supplementary file 1 [file ijerph-19-03860-s001.zip › ijerph-1606146-supplementary.pdf]

Supplementary Material

Table S1. Sources of data used in this study

Data category	Time	Unit	Source
Land use/cover	2000/2020	-	http://www.resdc.cn/
Climate scenario data	1961-2050	-	http://www.climatechange-data.cn/
potential cropland production	2000/2020	kg/ha	http://www.resdc.cn/
forest canopy density	2000/2020	-	http://earthenginepartners.appspot.com/
vegetation cover density	2000/2020	-	http://www.dsac.cn/
TEM	2000-2020	°C	http://www.resdc.cn/
PRE	2000-2020	mm	http://www.resdc.cn/
DEM	-	m	http://www.resdc.cn/
Slope	-	°	From DEM data
Geomorphic types	-	1-8	http://www.resdc.cn/
Sand	-	%	http://westdc.westgis.ac.cn
Silt	-	%	http://westdc.westgis.ac.cn
Clay	-	%	http://westdc.westgis.ac.cn
Drainage	-	1–7	http://westdc.westgis.ac.cn
PH	-	-log（H^+）	http://westdc.westgis.ac.cn
AWC	-	1–5	http://westdc.westgis.ac.cn
Soil type	-	1–14	http://westdc.westgis.ac.cn
Soil texture	-	1–5	http://westdc.westgis.ac.cn

Population density	2000/2020	10^4 persons/km^2	http://www.resdc.cn/
GDP	2000/2020	10^4 yuan/km^2	http://www.resdc.cn/
Vegetation	-	（1–13）	http://www.resdc.cn/
Crop production	2000-2020	10^4 t	https://data.cnki.net/Yearbook/
Livestock numbers	2000-2020	10^4 h	https://data.cnki.net/Yearbook/

Table S2. Growth rate of demands for land-use under different scenarios (%/year)

**Scenarios**	Crop production (t)	Livestock numbers (h)	Built-up land (km^2)	Forest land (km^2)	Ecological land (km^2)	Restricted areas
No LULC change	-	-	-	-	-	-
TREND	1.00%	0.30%	4.00%	0.20%	-	-
FOREST	1.00%	0.15%	4.00%	0.25%	-	-
CONSERVATION	1.00%	0.15%	4.00%	0.20%	0.05%	nature reserves

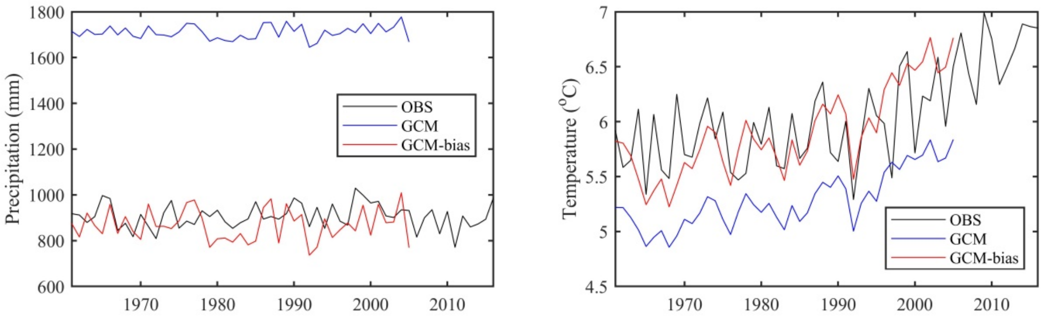

Figure S1. Comparison between observed precipitation and temperature before and after model revision on annual scale in the Hengduan Mountain region

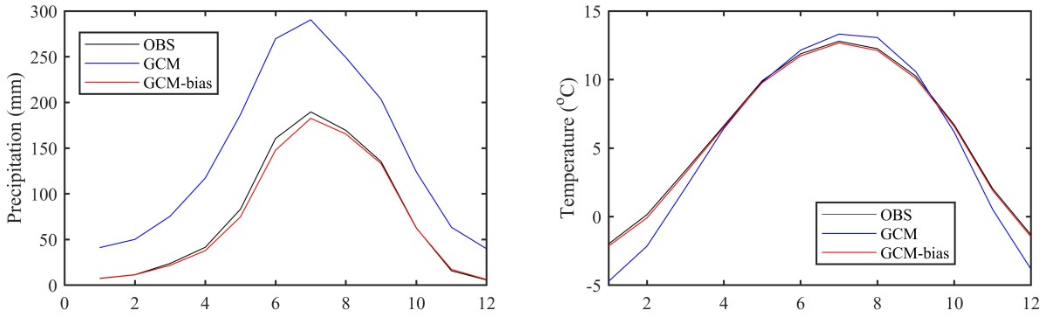

Figure S2. Comparison between observed precipitation and temperature before and after model revision on monthly scale in the Hengduan Mountain region

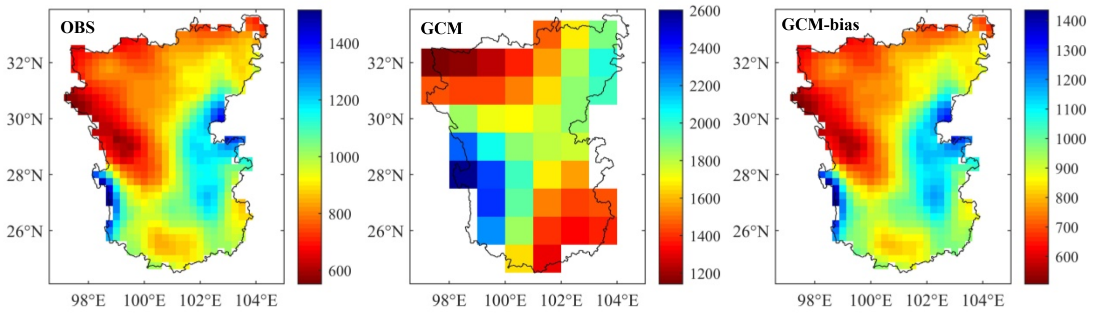

Figure S3. Comparison of spatial distribution of precipitation in the Hengduan Mountain region before and after model revision

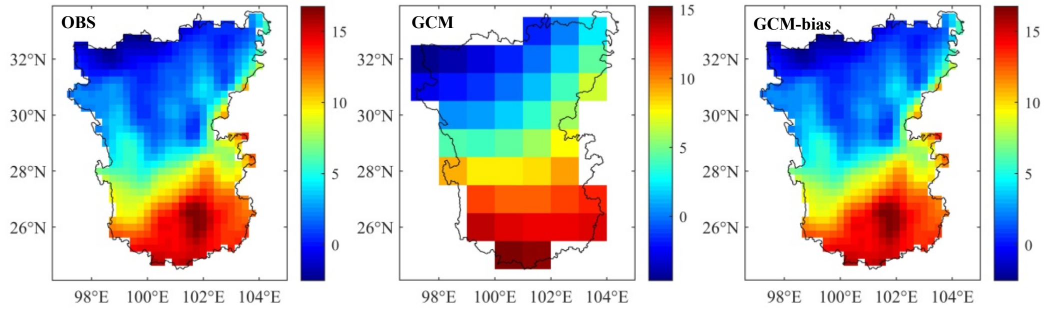

Figure S4. Comparison of spatial distribution of temperature in the Hengduan Mountain region before and after model revision
